# Detection of traumatic internal carotid artery pseudoaneurysm by postmortem imaging: A case report

**DOI:** 10.1097/MD.0000000000028544

**Published:** 2022-01-14

**Authors:** Lei Wan, Yanxiang Song, Zhengdong Li, Maowen Wang, Fengxiang Song, Jianhua Zhang, Donghua Zou, Ningguo Liu, Yuxin Shi, Zhiyong Zhang

**Affiliations:** a Department of Radiology, Shanghai Public Health Clinical Center, Fudan University, Shanghai, China; b Shanghai Key Laboratory of Forensic Medicine, Key Laboratory of Forensic Science, Ministry of Justice, Shanghai Forensic Service Platform, Academy of Forensic Science, Shanghai, China; c Institute for Regenerative Medicine, Shanghai East Hospital, School of Life Sciences and Technology, Tongji University, Shanghai, China.

**Keywords:** aneurysm, carotid artery, false, internal, postmortem angiography, postmortem imaging, postmortem MSCT/MRI, sphenoid sinus, traumatic, virtopsy

## Abstract

**Rationale:**

Postmortem imaging (PMI), including computed tomography (PMCT), postmortem computed tomography angiography (PMCTA), and postmortem magnetic resonance imaging (PMMRI), is rapidly becoming effective and a practical method in forensic medicine. This study aimed to present a specific forensic case in which the PMI approach and its applications were used.

**Patient concerns:**

A 40-year-old male patient had moderate unilateral nose bleeding constantly 10 times after suffering from a head injury induced by a car accident. After a bilateral massive nose bleeding for the last time, he died from hemorrhagic shock. Traumatic internal carotid artery pseudoaneurysm (TICAP) was suspected in this patient.

**Diagnosis, interventions, and outcomes:**

A whole-body scanning was performed using PMCT and PMMRI. Then, PMCTA using left ventricular cardiac puncture was also implemented. A water-soluble contrast agent was injected into the left ventricle and pumped toward the intracranial, followed by a repeated whole-body PMCT scan. The PMCT/PMMRI detected a high-density/signal mass inside the left sphenoid sinus. The PMCTA detected a distinct leakage of the contrast agent into the left sphenoid sinus from an adjacent aneurysm of the C3 section of the left internal carotid artery. Autopsy and histology confirmed a TICAP inside the sphenoid sinus.

**Lessons:**

This case showed that the PMI was of great value for identifying the cause of death in special cases. When vascular lesions are suspected in the body, PMI and especially the PMCTA approach may be an effective detection method.

## 1 Introduction

Traumatic internal carotid artery pseudoaneurysm (TICAP) is a rare complication of vascular injury after craniocerebral injury and accounts for approximately 0.5% of intracranial aneurysms. TICAP frequently occurs at the internal carotid-cavernous sinus and petrous bone segments, considering cranial base fracture as the main cause.^[1]^ Patients with TICAP present repeated epistaxis that is difficult to control, a condition that often leads to hemorrhagic shock or asphyxia, seriously threatening life. If it is misdiagnosed and mistreated as a general nosebleed treatment in clinical practice, the patients may die at any time due to uncontrollable bleeding.

The death cause of cadavers can be evaluated using postmortem imaging (PMI). PMI is a useful diagnostic tool for forensic cases of death from a medical dispute. However, this procedure is also described as virtopsy in Switzerland,^[2]^ virtual autopsy in France,^[3]^ radio-autopsy in Germany,^[4]^ and autopsy imaging (Ai) in Japan.^[5]^ Although the descriptions and concept of PMI in these countries differ somewhat, all involve the analysis of a cadaver using computed tomography (CT) and/or magnetic resonance imaging (MRI) to acquire postmortem medical information.

The minimally invasive autopsy with whole-body angiography is generally implemented as the routine coronial autopsy is complicated and time-consuming. Saunders et al^[6]^ proposed that targeted cardiac postmortem computed tomography (PMCT) could help overcome this problem. In previous studies, traumatic aortic rupture, cerebral arteriovenous malformation, and coronary heart disease were identified using PMCT and postmortem computed tomography angiography (PMCTA), and a whole-body angiographic approach was developed using left ventricular cardiac puncture.^[7–9]^ This study reported the case of TICAP that was not found during life but was newly recognized on PMI, especially in the PMCTA approach, using left ventricular cardiac puncture. Autopsy and histology confirmed a TICAP inside the sphenoid sinus. The present study was novel in exploring the possibility of TICAP in severe head injury using PMI comparison with pre-life imaging data.

## 2 Case description

### 2.1 Case report

**Figure 1:**
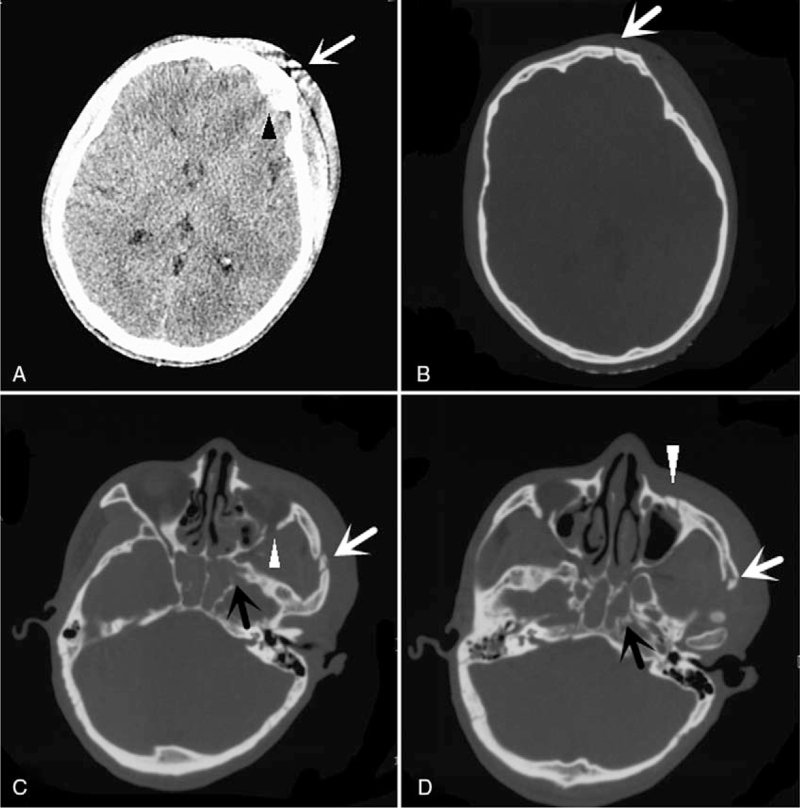
Pre-life CT findings. (**A**) Left frontotemporal epidural hematoma (black arrowhead) and left frontotemporal top scalp hematoma (white arrow). (B) Left frontal temporal bone fracture (white arrow). (C) Sphenoid sinus fracture and blood accumulation in sphenoid sinus (black arrow), left zygomatic arch fracture (white arrow), and fracture of lateral orbital wall (white arrowhead). (D) Sphenoid sinus fracture and blood accumulation in sphenoid sinus (black arrow), left zygomatic arch fracture (white arrow), and left anterior wall of maxillary sinus fracture (white arrowhead).

A 40-year-old, lean, muscular man was injured in a traffic accident and taken to hospital immediately. A head and facial CT examination was performed on the victim at the emergency clinic. The CT showed left frontotemporal epidural hematoma, frontal temporal bone fracture, middle cranial fossa (sphenoid sinus) and left anterior cranial fossa fractures with intracranial gas accumulation, blood accumulation in sphenoid sinus, left orbital wall fractures with gas accumulation, left zygomatic arch and anterior wall of maxillary sinus fractures, left eyelid hematoma, and left frontotemporal top scalp hematoma (Fig. 1).

The young man was given conservative treatment, including anti-inflammatory, rehydration, stomach protection, and nutrition brain cells. Five days after admission, sudden bleeding occurred from the left nasal cavity without obvious inducement, and the blood rushed out from the left nasal cavity, which was dark red. The effect of nasal compression was not good, and the bleeding was controlled by bilateral nasal packing and compression of the left cervical blood vessel. Symptoms were relieved after admission to the hospital for 1 week, and he was discharged thereafter.

However, he bled from the left nasal cavity 10 times for a period of 2 months after being discharged. In the last epistaxis, massive hemorrhage occurred from his both nasal cavities, and he lost consciousness. He was sent to the hospital immediately and died after emergency treatment. The clinically diagnosed cause of death was hemorrhagic shock.

The corpse was transported to the Academy of Forensic Science for further investigations 3 days after his death. The corpse was that of a strong young man with a great physique. Both eyelids and bulbar conjunctiva were pale, gauze packing was seen in both nasal cavities, double-cavity rubber balloon tube packing was seen in the right nasal cavity, and the nail beds of both fingers and toes were pale. No other abnormalities were found, and the cause of death and manner of the injury could not be determined by external examination.

### 2.2 PMMRI examination

PMMRI was carried out 1 hour after the external examination, using a 1.5-T clinical MR machine (Achieva, Philips Medical Systems, Best, The Netherlands) and examined using a SENSE torso coil without the use of contrast material. The corpse was placed in the supine position, with arms beside the body and was examined from head to pelvis. Standard sequences were coronal whole-body TIM T1-weighted imaging (T1WI) and short time inversion recovery sequences with a slice thickness of 5 mm; axial and sagittal images of the head, thorax, abdomen, and pelvis consisting of T1WI, T2W with fat saturation, T2-weighted imaging (T2WI), and T2WI with fluid-attenuated inversion recovery (FLAIR) sequences, all with a slice thickness of 5 mm.

### 2.3 PMCT and PMCTA examination

Postmortem magnetic resonance imaging (PMCT) was carried out 2 hours after the PMMRI examination. The entire body was scanned using a 40-slice multislice computed tomography (MSCT) system (Definition AS; Siemens Medical Solutions, Munich, Germany). The acquisition of raw data was achieved using the following settings: voltage, 120 kV; current, 240 mA; and collimation, 6.0 × 1.0 mm. Image reconstruction was achieved at slice thicknesses of 5 and 0.625 mm, each with an increment of half the slice thickness–, soft tissue–, and bone-weighted reconstruction kernel. Image review and 3D reconstructions were carried out on a CT workstation (Syngo Imaging XS; Siemens Medical Solutions) and Mimics 14.0 (Materialise Inc., Leuven, Belgium).

**Figure 2:**
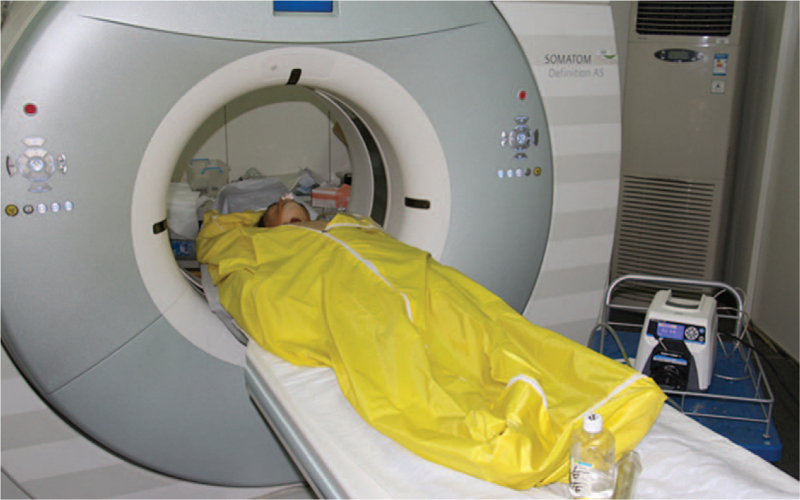
Targeted PMCTA of the right internal carotid artery was performed. The device shown on the right is a special pump used to inject the contrast agent.

During PMCTA, one end of the customized angiographic device^[10]^ was inserted into the right internal carotid artery and fixed in place (targeted PMCTA). PMCTA was performed at a flow rate of approximately 100 mL/min in continuous perfusion mode (Fig. 2). Then, 1000 mL of contrast media [diatrizoate meglumine and normal saline (0.9%) at 1:2 ratio] was injected. An MSCT scan was performed directly after administration of the contrast media solution.

### 2.4 Autopsy

A traditional autopsy was performed about 1 hour after the PMCTA examination. Blood taken by cardiac puncture was sent for toxicological analysis before the contrast agent injection in the PMCTA procedure. During autopsy, external and internal examinations of the body were performed. The sphenoid body was opened under the guidance of PMCT results, and the C3 segment of the left internal carotid artery was taken out. Histology samples of most of the organs within the cranial, thoracic and abdominal cavities were taken and processed with hematoxylin and eosin staining.

## 3 Results

**Figure 3:**
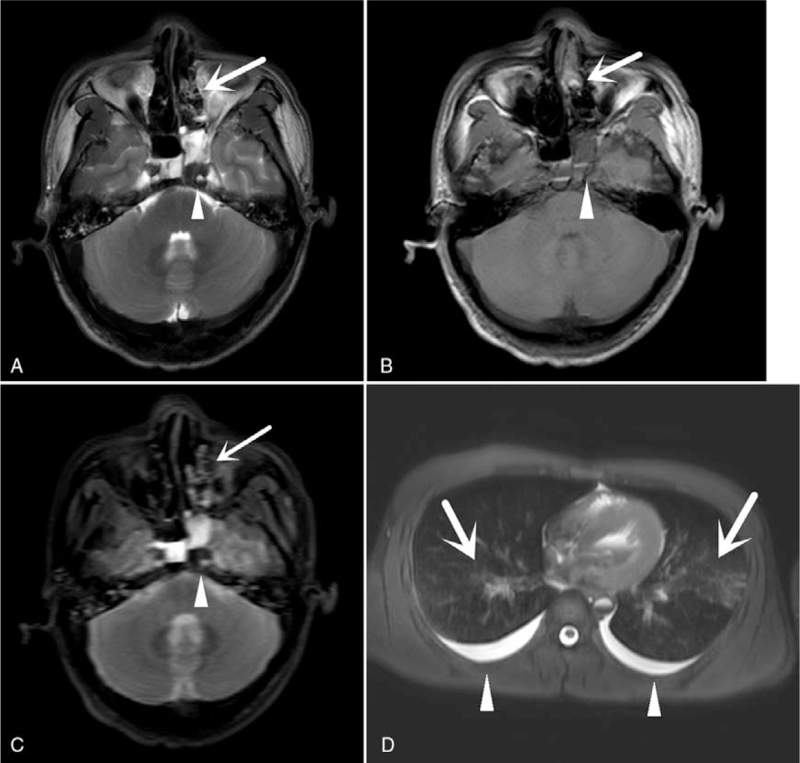
PMMRI findings. (**A–C**) T2WI, T1WI, and T2W FLAIR images showing hemorrhage in the sphenoid sinus (white arrowhead) and nasal cavity (white arrow). (D) T2WI showing a small amount of pleural effusion on both sides (white arrowhead) and some congestion of both lungs (white arrow).

**Figure 4:**
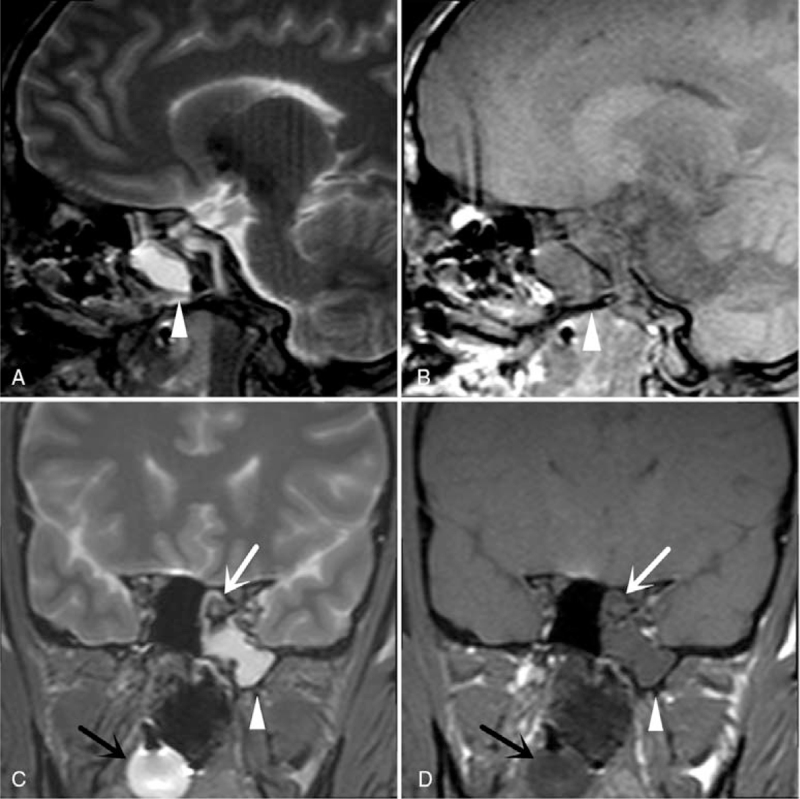
PMMRI findings. (A and B) T2W and T1W sagittal position images showing hemorrhage in the sphenoid sinus (white arrowhead). (C and D) T2W and T1W coronal position images showing hemorrhage in the sphenoid sinus (white arrowhead), intracranial tissue–like pseudoaneurysm protrusion into the sphenoid sinus (white arrow), and a balloon in the nasal cavity (black arrow).

During PMMRI, hemorrhage in the sphenoid sinus and nasal cavity was detected. A balloon was expanded in the nasal cavity. Intracranial tissue–like pseudoaneurysm protruding into the sphenoid sinus was also observed. A small amount of pleural effusion was also revealed on both sides. Some congestion of both lungs was also observed (Figs. 3 and 4).

**Figure 5:**
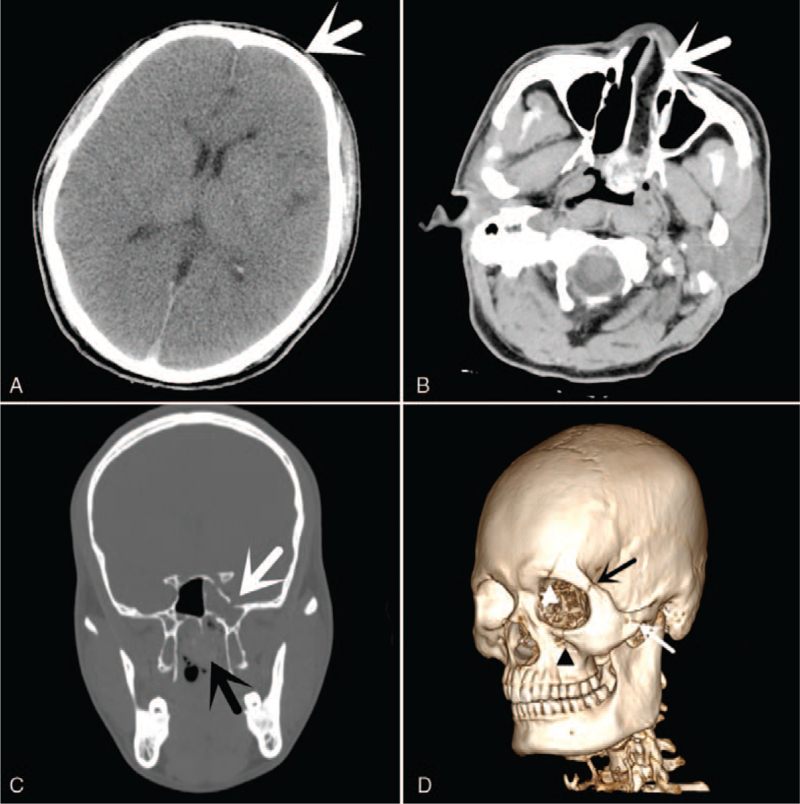
PMCT findings. (A) Scalp hematoma and epidural hematoma were absorbed (white arrow). (B) Hemorrhage in the nasal cavity (white arrow). (C) PMCT coronal multiplanar reconstructions. Sphenoid sinus fracture and bone defect, hemorrhage in the sphenoid sinus (white arrow), and hemorrhage in the nasal cavity (black arrow). (D) Three-dimensional reconstruction of PMCT. Old fractures of left lateral orbital wall (black arrow), left zygomatic arch (white arrow), frontal temporal bone (white arrowhead), and left anterior wall of maxillary sinus (black arrowhead).

During PMCT, the scalp hematoma and epidural hematoma were found to be absorbed compared with the CT images before death. Hemorrhage in the sphenoid sinus and nasal cavity were also detected. Sphenoid sinus fracture and bone defect were revealed. Old fractures of left lateral orbital wall, left zygomatic arch, frontal temporal bone, and left anterior wall of maxillary sinus were also observed (Fig. 5).

**Figure 6:**
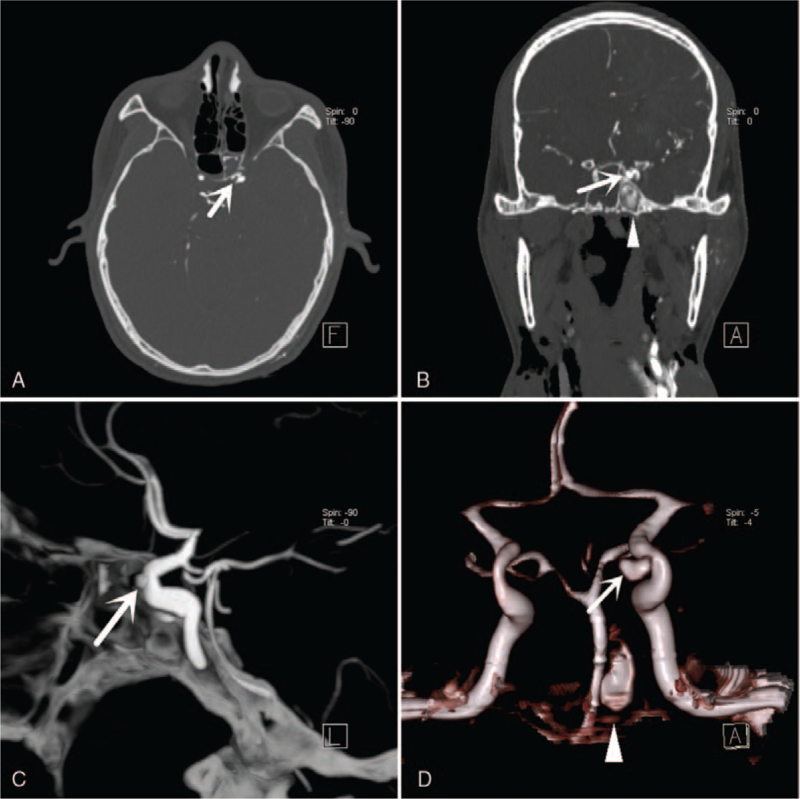
PMCTA findings. (A) Axial image showing a pear-shaped aneurysm of the C3 section of the left internal carotid artery (white arrow). (B) Coronal image showing a pear-shaped aneurysm of the C3 section of the left internal carotid artery (white arrow) and a distinct leakage of contrast agent into the left sphenoid sinus (white arrowhead). (C) PMCTA sagittal MIP images showing a pear-shaped aneurysm of the C3 section of the left internal carotid artery (white arrow). (D) Three-dimensional reconstruction of the PMCTA showing a distinct leakage of the contrast agent into the left sphenoid sinus (white arrowhead) from an adjacent pear-shaped aneurysm of the C3 section of the left internal carotid artery (white arrow).

**Figure 7:**
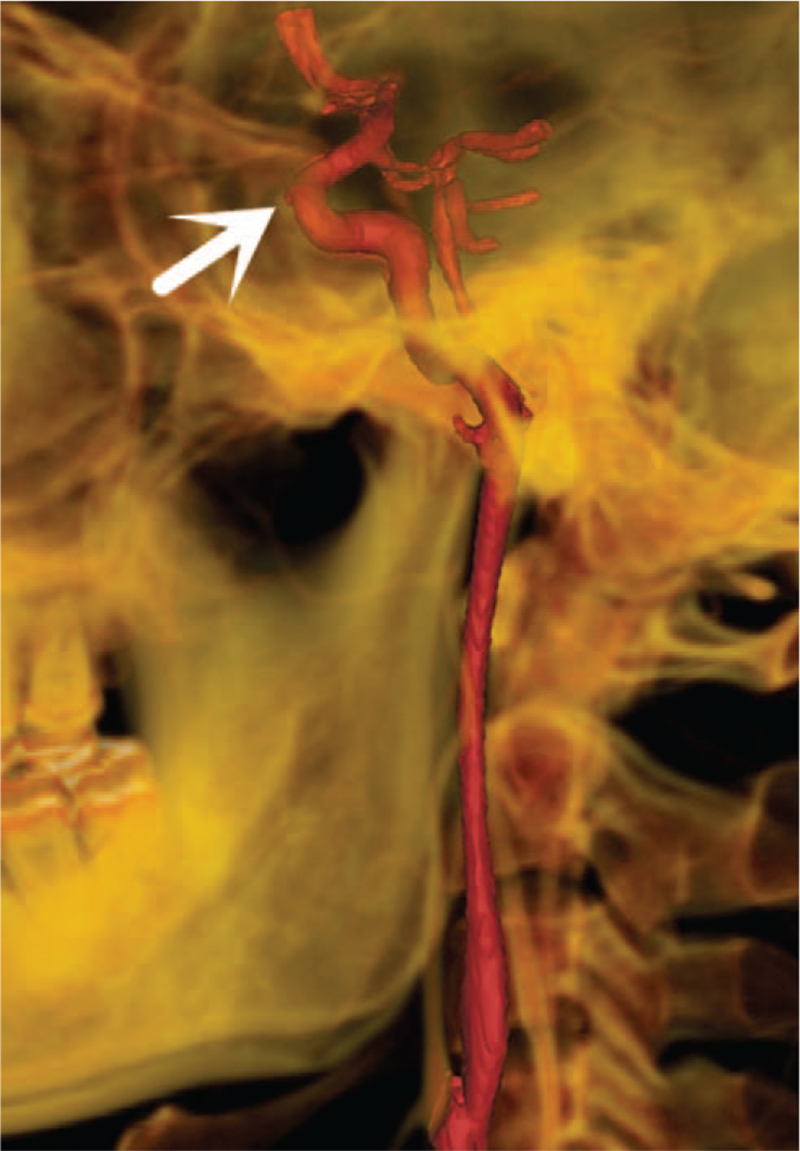
Three-dimensional reconstruction of the PMCTA using Mimics 14.0 showing a pear-shaped aneurysm of the C3 section of the left internal carotid artery (white arrow).

During PMCTA, a distinct leakage of contrast agent into the left sphenoid sinus from an adjacent pear-shaped aneurysm of the C3 section of the left internal carotid artery was detected (Figs. 6 and 7).

**Figure 8:**
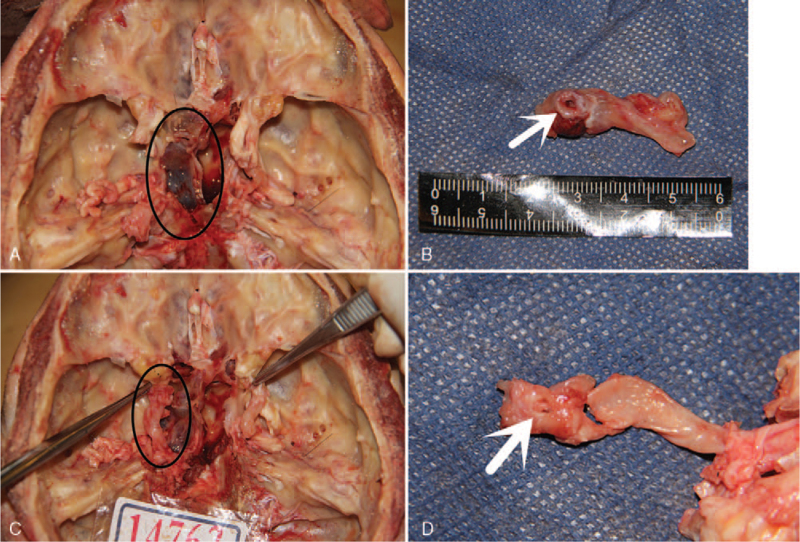
Autopsy findings and procedure. (A and B) A rupture pouch-like structure, pale and inflamed tissue at the breach, and blood accumulation and clot attachment on the front side of the left sphenoid sinus. (C and D) A pedicle-like structure extending into the left sphenoid sinus in the C3 segment (siphon curve) of the left internal carotid artery.

During the autopsy, a pedicle-like structure extended into the left sphenoid sinus in the C3 segment (siphon curve) of the left internal carotid artery, a rupture pouch-like structure was observed, the tissue at the breach was pale and inflamed, and blood accumulation and clot attachment occurred on the front side of the left sphenoid sinus (Fig. 8).

**Figure 9:**
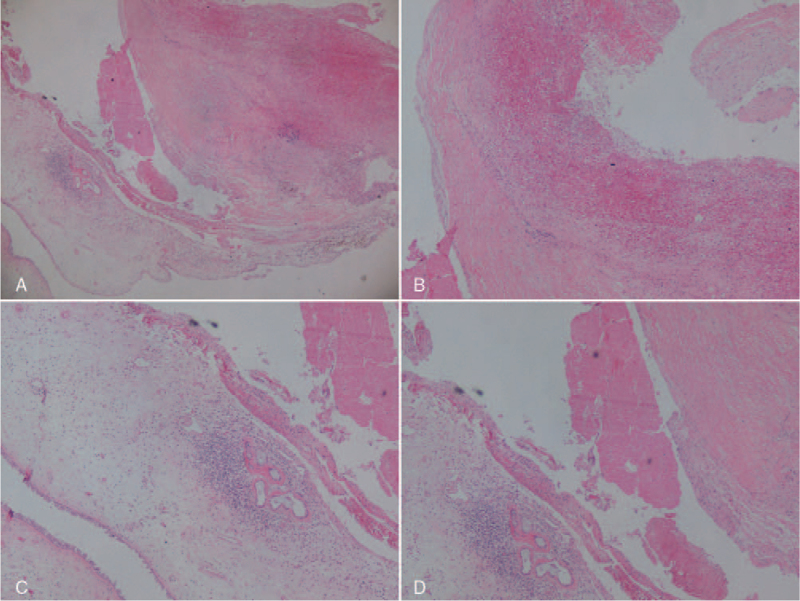
Histological examination results. (A) General view of the aneurysm and sphenoid sinus mucosa. (B) Chronic inflammation, bleeding, and thrombosis of the aneurysm wall. (C) Sphenoid sinus mucosa edema and inflammation. (D) Thrombosis in the aneurysm cavity.

The histological examination revealed a large amount of fibrous tissue proliferation and cystic structure formation under the sphenoid sinus mucosa, multiple speckle hemorrhages in the cyst wall with inflammatory changes, thrombus attachment and shedding in the cyst wall and part of the cyst cavity, and no elastic fibers in the cyst wall and smooth muscle tissue. Local internal carotid artery rupture and shedding of the intima, fibrous tissue proliferation under the intima, scattered old necrosis with calcification foci on the tube wall, and local necrotic tissue and mucus-like material accumulation with inflammatory cell infiltration were also noted (Fig. 9).

## 4 Discussion

Injuries of the intracranial segment of the internal carotid artery often occur in the C3–C4 segment of the internal carotid artery (Fischer classification), which is related to the susceptibility to fracture of the anterior skull base during trauma. The C4 segment injury often leads to an internal carotid artery–cavernous sinus fistula, while the C3 segment injury results in a pseudoaneurysm called TICAP.^[11]^ The formation of TICAP occurs mainly due to the rupture of the internal carotid artery C3 segment caused by trauma. Blood overflows into the sphenoid sinus through the rupture of the internal carotid artery and the sphenoid sinus fracture. The bleeding is wrapped by surrounding tissues as a localized hematoma. The blood in the hematoma rapidly coagulates into a blood clot, the rupture of the artery wall is temporarily blocked, a part of the blood clot is liquefied and dissolved in the later stage, and a cavity appears in the hematoma. The vascular rupture is connected with the hematoma due to the continuous impact of the arterial pulse, and the arterial blood is re-injected into the hematoma cavity, leading to fluctuations at the same time. The periphery of the hematoma is surrounded by fibrous tissue that gradually becomes hyperplasic, forming a pseudoaneurysm at last. Although TICAP is rare in clinical practice, its complications are serious, and patients can die from shock due to nasal hemorrhage; therefore, adequate attention should be paid.^[12]^ TICAP mainly showed the image of pear-shaped aneurysms in the sphenoid sinus. This type of aneurysm has no aneurysm wall. It is the organization of hematoma or the formation of a capsule in the sphenoid sinus mucosa. Under the continuous impact of blood, the aneurysm expands and thins, and finally ruptures. Blood enters the nasal cavity from the sphenoid sinus through the ethmoid sinus, resulting in a large amount of epistaxis, which may result in death due to shock.^[13,14]^

In the present case, PMI examination showed that the sphenoid bone fracture was accompanied by bone loss. The C3 segment of the left internal carotid artery showed an oval tumor-like structure and communicated with the adjacent sphenoid sinus. After angiography, the contrast agent was missed into the sphenoid sinus, indicating an aneurysm and rupture. We needed to consider whether this was a true aneurysm or a traumatic pseudoaneurysm and whether a causal relationship existed with the traffic accident.

The difference between a true and a false aneurysm (pseudoaneurysm) is the lack of all 3 layers of the arterial wall in a pseudoaneurysm compared with a true aneurysm, which presents as a dilation of an intact arterial wall. The trauma can cause a disruption of the vessel wall, with extravasation of the blood and formation of a hematoma and pseudocapsule that can expand from the pressure of the blood flow. Congenital aneurysms generally have no history of head trauma, while traumatic aneurysms have a history of severe head trauma, often accompanied by skull fractures.^[15]^

In the case reported in this study, considering a craniocerebral trauma during his lifetime, especially with a skull base fracture, no elastic fibers and smooth muscle tissue were seen in the aneurysm wall during the later period. The formation mechanism was that the internal carotid artery close to the sphenoid sinus was directly damaged by the traumatic sphenoid sinus fracture. The internal artery was connected to the sphenoid sinus through the fracture of the sphenoid sinus and finally formed a pseudoaneurysm. In addition, the pathological changes of other fatal diseases were not detected in this case, and the forensic pathological changes of mechanical asphyxia were not found. Therefore, the cause of death was believed to be a TICAP. Under the continuous impact of blood, the aneurysm expanded and thinned, and eventually ruptured, resulting in shock and death.

In the present case, several imaging techniques were combined, each with its own advantages and complementary to each other. Considering that the main injuries were probably in the aorta, carrying out a PMCTA through the right internal carotid artery (targeted PMCTA) rather than through arteries in the lower limbs was more purposeful, effective, and time saving. PMCTA images revealed distinct evidence of aortic rupture in consequence. The advantages of the present approach were that it was targeted, convenient, and time saving. However, it is effective only in some specific cases, such as angiorrhexis in aneurysm.

Cranial base fracture and epistaxis were previously considered as the primary manifestations of TICAP.^[16]^ If a skull base fracture is observed on CT examination after trauma with nose bleeding, CTA and digital subtraction angiography should be performed as soon as possible to confirm whether a traumatic aneurysm is present. The time to first clinical presentation of artery dissection can vary from hours to years after trauma. In the majority of cases, epistaxis occurs within 1 to 3 months of injury, although delays in the presentation of up to 40 years have been seen.^[17]^ In the present case, had traumatic aneurysm been recognized early and endovascular interventional treatment given timely, including detachable balloon, micro-coil, endovascular stent, and so forth,^[18–20]^ then it would not have resulted in death.

## Author contributions

**Funding acquisition:** Maowen Wang.

**Methodology:** Fengxiang song.

**Project administration:** Zhengdong Li, Jianhua Zhang.

**Resources:** Donghua Zou, Ningguo Liu.

**Supervision:** Zhiyong Zhang.

**Writing – original draft:** Lei Wan, Yanxiang Song.

**Writing – review & editing:** Yuxin Shi.
